# Hematochezia caused by tandospirone in a patient with major depressive disorder and anxious distress: a case report

**DOI:** 10.3389/fpsyt.2023.1209354

**Published:** 2023-07-17

**Authors:** Xingmei Jiang, Zhongrui Ma, Zhixiong Li, Ying Ou, Zhenhua Luo, Zhe Li

**Affiliations:** ^1^Mental Health Center, West China Hospital, Sichuan University, Chengdu, Sichuan, China; ^2^Sichuan Clinical Medical Research Center for Mental Disorders, Chengdu, Sichuan, China; ^3^Geriatric Diseases Institute of Chengdu, Department of Psychosomatics, Chengdu Fifth People’s Hospital, Chengdu, Sichuan, China; ^4^The Third Department of Clinical Psychology, Karamay Municipal People’s Hospital, Karamay, Xinjiang, China; ^5^The First Affiliated Hospital of Traditional Chinese Medicine, Chengdu Medical College, Chengdu, Sichuan, China; ^6^Xindu Hospital of Traditional Chinese Medicine, Chengdu, Sichuan, China

**Keywords:** depressive disorder, anxious distress, tandospirone, antidepressant, hematochezia, case report

## Abstract

**Background:**

Major depressive disorder (MDD) with anxious distress is a relatively common condition that is often associated with a poor treatment response. In order to enhance the effectiveness of MDD treatment, 5-HT1A agonists like tandospirone are often prescribed in conjunction with antidepressants. While it is known that antidepressants can increase the risk of bleeding, whether tandospirone poses a similar risk remains uncertain.

**Case presentation:**

We presented the case of a 55-year-old Chinese woman diagnosed with MDD and anxious distress. After receiving various types of antidepressants, she experienced hematochezia following the administration of tandospirone, sertraline, and agomelatine. The occurrence of hematochezia ceased after tandospirone was discontinued. The patient was subsequently discharged with a treatment regime consisting of sertraline and agomelatine. During the 1-month follow-up, she reported no hematochezia.

**Conclusion:**

Tandospirone may potentially increase the risk of hematochezia in patients with MDD and anxious distress.

## Background

Anxiety disorder involves feelings of tension and being threatened, restlessness, irritability, sleep disturbance, palpitations, dry mouth, and sweating (1). The lifetime prevalence of anxiety disorder is estimated at 3.7% worldwide (2) and 6.1% in China (3). First-line pharmacotherapies for anxiety disorders are generally considered to be selective serotonin reuptake inhibitors and serotonin norepinephrine reuptake inhibitors (4), although benzodiazepines and 5-HT1A receptor agonists are also commonly prescribed (5). The 5-HT1A receptor agonist tandospirone demonstrates comparable anxiolytic efficacy to benzodiazepines (5), while presenting a lower risk of dependence and fewer adverse effects on sleep quality and cognition (6–8). The absence of significant side effects likely stems from tandospirone’s highly selective affinity for the 5-HT1A receptor, along with weak or negligible affinity for dopaminergic, noradrenergic, cholinergic, and GABAergic receptors (7).

Tandospirone is also often combined with antidepressants to treat major depressive disorder (MDD) comorbid with anxiety (5, 9–12). It is worth noting that up to 75% of patients with MDD also experience anxiety (13, 14). The medications most often used to treat anxious depression include antidepressants such as selective serotonin or noradrenaline reuptake inhibitors, as well as other medications that operate through different mechanisms, such as bupropion, ketamine, and brexpiprazole (15). The combination of antidepressants and anxiolytics has the potential to enhance the overall therapeutic effects of antidepressants in patients with MDD and anxious distress (9). Considering the side effects associated with benzodiazepines, clinicians often employ a combination of antidepressants and 5-HT1A agonists to treat patients with MDD and anxious distress (15–19).

Hematochezia, characterized by the presence of blood in the stool, is the most common clinical manifestation of lower gastrointestinal bleeding. Common causes of this condition include diverticular bleeding, anorectal disease such as hemorrhoidal bleeding, and various types of colitis (20). Risk factors associated with such bleeding include smoking, alcohol consumption, the use of nonsteroidal anti-inflammatory medications or antiplatelet medications, and old age (20, 21). The majority of individuals who experience lower gastrointestinal bleeding are elderly (22), often have underlying medical conditions, and are receiving antiplatelet or anticoagulant treatment (22). In some cases, lower gastrointestinal bleeding can escalate to a massive extent (23).

The fact that antidepressants can increase bleeding risk is well established (24). However, it remains unclear whether tandospirone increases the bleeding risk among patients with MDD and anxious distress. Here, we describe the case of a 55-year-old Chinese woman with anxious depression who developed hematochezia, a common symptom of lower gastrointestinal bleeding, after taking a combination of tandospirone and antidepressants. The hematochezia ceased 1 day after discontinuation of tandospirone. This case highlights the potential bleeding risk associated with tandospirone for patients with anxiety and depression.

### Case presentation

A 55-year-old Chinese woman presented to our hospital with recurring nervousness, worry, and depression. Over the past year, she had been worried about the progression of previously diagnosed lung nodules to lung cancer, the possibility of brain atrophy, and the development of Alzheimer’s disease, similar to her father. She also experienced worry over trivialities matters. The patient reported numbness in her hands, palpitation, dizziness, and poor sleep, leding her to take sleeping pills. She described her mental and physical discomfort as nearly constant throughout the day. Initially, she sought medical help at a local hospital, where she was prescribed estazolam. She reported that estazolam improved her sleep and alleviate her physical discomfort. However, after 1 month, she experienced palpitation and precordial discomfort once again, prompting her readmission to the same local hospital. Despite undergoing coronary angiography and other examinations, no obvious abnormalities were detected.

During this period, while still using estazolam, the patient consistently expressed feeling of sadness and an inability to experience happiness, which intensified during the winter season. She had lost interest in engaging in activities and exhibited reluctance to form social connections or communicate with family members. The patient experienced overall fatigue, emotional numbness, difficulty concentrating, memory impairment, decreased appetite, and recurring thoughts that she would become a burden to her family members. Notably, she denied any self-injury, suicidal ideation, or negative self-evaluation. The patient visited a local hospital, where she receiving a diagnosis of generalized anxiety disorder. She was given the treatment of escitalopram oxalate (20 mg daily), alprazolam (0.4 mg at night), and olanzapine (5 mg at night), resulting in the complete alleviation of her symptoms, as reported by the patient.

After one month on this treatment, the patient underwent sinusitis surgery at a local hospital. Subsequently, she started experiencing nasal congestion and various discomforts, which triggered feeling of nervousness and restlessness. Additionally, she developed recurring concerns about the possibility of developing nasopharyngeal carcinoma. To address escalating symptoms, the dosage of alprazolam was adjusted to 0.2 mg in the morning, 0.2 mg at noon, and 0.4 mg at night. The patient reported experiencing complete relief from her anxious symptoms with this adjusted medication regimen.

After 5 months on the new treatment regime, the patient began experiencing symptoms such as numbness in the hands, palpitation, dizziness and depressive feeling again. Additionally, her sleep quality deteriorated. The local hospital adjusted her medication to fluoxetine (20 mg daily), alprazolam (0.4 mg three times a day), and olanzapine (5 mg at night). The patient reported improvements in her mood and sleep, although she continued to experience nervousness and worry. Concerned about the potential for addiction, the patient’s family decided to reduce the dosage of alprazolam. However, this adjustment exacerbated her feelings of nervousness and worry. As a result, the local hospital modified her treatment regime once again, prescribing fluoxetine (40 mg daily) and olanzapine (5 mg at night).

After 6 months on this regime, the patient continued to experience persistent feeling of worry, nervousness, palpitation, and restlessness. Therefore, she was referred to our hospital for further evaluation and management. It is important to note that she did not report any episodes of elation, excited talking, hallucinations, or delusions during the year before admission to our hospital.

### History of past illness

Over 30 years prior to the patient’s admission to our hospital, and 1 year before admission, she underwent surgery procedures for sinusitis. It is important to note that the patient and her family members denied any history of smoking, alcohol consumption, or use of psychoactive substances.

### Family history

The patient provided information that her father is currently alive and has been diagnosed with Alzheimer’s disease. Additionally, patient’s mother, sister, brother, uncle and aunt passed away due to lung cancer.

### Physical and laboratory examinations

Neurological examination results were unremarkable. Laboratory assays reveled a normal platelet count of 202 × 10^9^/L (reference range: 100–300 × 10^9^/L). However, there were elevated levels of triglycerides (2.04 mmol/L; reference range: 0.29–1.83 mmol/L), cholesterol (6.29 mmol/L; reference range: 2.80–5.70 mmol/L), low-density lipoprotein cholesterol (4.12 mmol/L; reference range: <4.0 mmol/L), fibrin and fibrinogen degradation products (6.4 mg/L; reference range: <5 mg/L), and D-dimer (2.80 mg/L FEU; reference range: 0.55 mg/L FEU). Routine assays for glycosylated hemoglobin, hormones, tumor markers and functional indicators of the thyroid, liver, and kidney did not reveal any obvious abnormalities. Routine tests of stool and occult blood in stool were normal. Color Doppler flow imaging indicated the presence of posterior tibial vein thrombosis. Computed tomography of the chest showed a few mixed ground-glass nodules, likely indicating inflammatory nodules in the middle lobe of the right lung. Additionally, calcification of the left coronary artery wall and multiple cysts in the liver were observed. Magnetic resonance imaging of the head revealed an ischemic lesion in the left temporoparietal junction. Ultrasonography suggested the presence of fatty liver, a hepatic cyst, a solid mass (possibly fibroids) in the uterus, and a nodule (potentially nodular goiter) in the right lobe of the thyroid. Conventional echocardiography demonstrated normal left ventricular systolic function but identified a left coronary pulmonary fistula and mild aortic regurgitation, which may suggest the presence of congenital heart disease.

### Further diagnostic work-up

The patient underwent a comprehensive psychiatric evaluation conducted by an experienced psychiatrist. The psychiatrist utilized the Chinese versions of the 24-item Hamilton Depression Scale, on which she scored 32, indicating moderate depression; and the Hamilton Anxiety Scale, on which she scored 34, indicating severe anxiety. She scored 26 on the Chinese version of the Mini-mental State Examination and 25 on the Chinese version of the Montreal Cognitive Assessment, indicating no obvious cognitive impairments.

### Final diagnosis

The patient expressed concerns regarding the possibility of developing lung cancer, brain atrophy or Alzheimer’s disease, leading to consideration of illness anxiety disorder as a differential diagnosis. However, it was noted that despite her worries about various aspects of life, she did not exhibit excessive health-related behaviors or maladaptive avoidance behaviors, even in the presence of a family history of Alzheimer’s disease and lung cancer. Additionally, she denied experiencing specific symptoms associated with lung cancer or Alzheimer’s disease. Instead, she reported symptoms such as numbness in the hands, palpitation, dizziness, and poor sleep. Taking into account these reported symptoms, as well as her depressive symptoms, the patient was diagnosed with recurrent moderate MDD with anxious distress, based on the criteria outlined in the 5th edition of the *Diagnostic and Statistical Manual of Mental Disorders* (25).

### Treatment

During the patient’s hospital stay, she received the following medications: lorazepam (0.5 mg at night), sertraline (50 mg daily), agomelatine (25 mg at night), tandospirone (10 mg three times a day). Additionally, the patient consistently received atorvastatin (20 mg at night as calcium tablets) to manage her hyperlipidemia. After 4 days on the same treatment regimen, the patient complained of hematochezia, which she reported never having experienced. To investigate the cause of the hematochezia, we assayed her coagulation function. The results showed a normal thrombocytes count (212; reference range: 100–300), but elevated levels of fibrin and fibrinogen degradation products (8.8 mg/L; reference range: <5 mg/L) and D-dimer (4.94 mg/L FEU; reference range: <0.55 mg/L FEU). We sought consultation with colleagues in the hematology department, who advised us to continue monitoring the patient’s condition. Occult blood was detected in stool, but upon performing a digital rectal examination, we did not identify any hemorrhoids or anal fissures. Although a colonoscopy was recommended, the patient declined the procedure. Considering the potential association between the medication regimen and the hematochezia, we decided to discontinue tandospirone and instead continue with lorazepam (0.5 mg at night), sertraline (50 mg daily), agomelatine (25 mg at night). After 1 day on the modified treatment, the patient reported that the hematochezia had ceased.

After 15 days on a treatment regime of sertraline (150 mg daily), agomelatine (25 mg at night) combined with physiotherapy, the patient’s condition improved significantly. Upon discharge, she reported experiencing significantly reduced level of anxiety and depression. She scored 8 on the Hamilton Depression Scale and 9 on the Hamilton Anxiety Scale. She scored 5 on the Chinese version of the Naranjo Causality Scale (26), indicating a probable adverse reaction to tandospirone, further supporting the decision to discontinue its use.

### Outcome and follow-up

**Figure 1:**
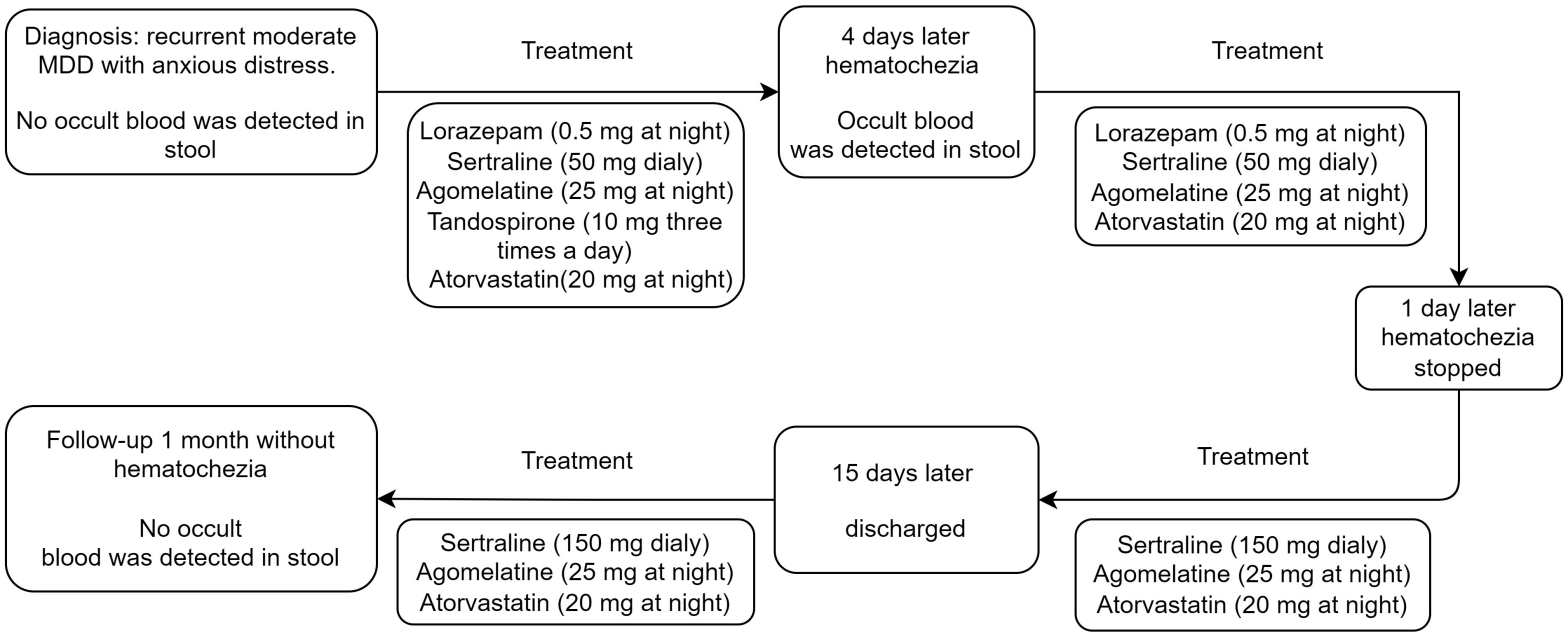
Timeline of treatment and the occurrence of the adverse reaction.

At the 1-month follow-up, the patient’s physical and mental condition remained stable, with no recurrence of hematochezia or signs of adverse medication effects. The patient reported good compliance with the medication regimen and expressed overall tolerability of the prescribed medications. No occult blood was detected in stool during this period. She scored 5 on the Hamilton Depression Scale and 6 on the Hamilton Anxiety Scale. Additionally, the patient reported experiencing an increased interest in engaging in her favorite leisure activities. Timeline of treatment and the occurrence of the adverse reaction is shown in Figure 1.

## Discussion

After diagnosing the patient with MDD with anxious distress, we initiated a combination of the selective serotonin reuptake inhibitor sertraline, which has proven effective against anxious depression (15); tandospirone, which can improve outcomes in patients on selective serotonin reuptake inhibitors (9, 27); and agomelatine, which can help improve sleep quality and reduce seasonal mood changes (28).

Hematochezia is the most common manifestation of lower gastrointestinal bleeding (22). However, in the case of our patient, there were no indications of platelet-related disease, anemia, leukemia, hemophilia or other hematological disorders that could lead to coagulation dysfunction. Additionally, there was no evidence of cirrhosis or hepatitis. Although the patient exhibited elevated levels of thrombocytes, D-dimer, fibrin and fibrinogen degradation product, which may reflect posterior tibial vein thrombosis, it is unlikely that clotting dysfunction was the cause of her hematochezia. The patient did not have a history of abnormal bleeding and was not taking antiplatelet medications such as aspirin or warfarin, anticoagulants such as heparin or low-molecular-weight heparin, or thrombolytic medications such as alteplase (29). Additionally, she reported no history of smoking or alcohol consumption. All these medications and behaviors are associated with an increased risk of lower gastrointestinal bleeding (20).

Instead, several factors that support attributing the patient’s hematochezia to tandospirone. First, the patient reported no prior use of tandospirone and no history of bleeding episodes. The onset of hematochezia occurred following the combined administration of sertraline, agomelatine and tandospirone, and the cessation of tandospirone resulted in the prompt resolution of the hematochezia. Second, the patient scored 5 on the Chinese version of the Naranjo Causality Scale, indicating a probable adverse reaction to tandospirone. Additionally, occult blood was detected in stool, further supporting the presence of hematochezia. Third, while selective serotonin reuptake inhibitors, including sertraline, are known to increase the risk of bleeding (30), it is unlikely that sertraline was the cause of the hematochezia in this case. The patient did not report hematochezia while taking escitalopram oxalate or fluoxetine before admission to our hospital, and there were no instance of hematochezia when the patient was taking sertraline without tandospirone during her hospital stay or at the 1-month follow-up. Moreover, bleeding induced by sertraline tends to affect the upper gastrointestinal tract, presenting as black, tarry stools, vomiting of blood, or abdominal pain, rather than hematochezia (31). Fourth, while agomelatine can increase the risk of bleeding (31), the associated risk may be small and does not appear to impact platelet aggregation or function (32).

During the treatment of our patient, we took into consideration the possibility of medication-medication interactions, particularly in relation to serotonergic antidepressants, which have been associated with an increased risk of bleeding (31). The underlying cause of these adverse reactions is believed to be the reduction of serotonin reuptake by platelets (31, 33). Serotonin plays a role in platelet aggregation induced by adenosine diphosphate, epinephrine, and collagen (34). Enteroendocrine cells, which are specialized cells of the intestinal tract, secrete a wide variety of neuroendocrine transmitters such as serotonin (35). Tandospirone, with its highly selective affinity for 5-HT1A receptors (36) and downregulation of postsynaptic 5-HT2 receptors (37), shares a similar mechanism of action with buspirone, which has been associated with rectal bleeding (38). Sertraline inhibits the reuptake of serotonin and acts as a 5-HT1A agonists. Combining tandospirone with sertraline could potential further inhibit platelet aggregation, thereby increasing the risk of bleeding. It is important to note that tandospirone is primarily metabolized by the 3A4 isoform of cytochrome P450 (39), while sertraline, at high doses, can inhibit the 2D6 isoform of cytochrome P450 (40). Therefore, in this case, we do not suspect a cytochrome P450-mediated interaction between tandospirone and sertraline.

We hypothesize that the combination of tandospirone with sertraline may lead to an excessive increase in the concentration of 5-hydroxytryptamine in the synaptic cleft. This excessive increase in serotonin levels may potentially inhibit platelet aggregation, thereby increasing the risk of lower gastrointestinal bleeding. Further study is needed to investigate the specific mechanisms by which tandospirone could increase the risk of hematochezia and whether it can interact with other medications to exacerbate this risk.

Taking into account the patient’s perspective is part of an integrated approach to treatment optimization (41). Our patient experienced significant anxiety when she developed hematochezia after using anti-depressants and tandospirone. This unexpected side effect led her to express a desire to discontinue all medicines. However, we took the time to explain the nature and causes of her condition, MDD with anxious distress, as well as the potential risks and benefits of the prescribed medications. We also provided information on how patients can cope with side effects. By addressing her concerns and ensuring she had a comprehensive understanding of her condition and treatment, we were able to alleviate her worries. As a result, the patient reported satisfaction with the treatment plan and demonstrated good compliance. This experience highlights the importance of the physician’s role as a detective, advisor, and collaborator (42), actively involving the patient in the decision-making process and considering their perspective. Taking into account the patient’s viewpoint is particularly crucial in mental health care, where individual experiences and preferences play a significant role in the success of treatment outcomes.

## Conclusion

Clinicians should keep in mind that tandospirone, especially when combined with antidepressants, may increase the risk of hematochezia in patients with MDD and anxious distress. If such bleeding occurs, clinicians should immediately assess the possibility of an adverse reaction and consider discontinuing tandospirone. This may help ensure patient safety and thereby improve satisfaction and treatment compliance.

## Data availability statement

The original contributions presented in the study are included in the article/supplementary material, further inquiries can be directed to the corresponding authors.

## Ethics statement

Written consent was obtained from the participant for the publication of this case report.

## Author contributions

YO collected the patient data, which ZM and ZxL analyzed. XJ wrote the manuscript together with other authors and revised it based on input from all authors. ZL and ZhL contributed to the conception and design of the work. All authors read and approved the final manuscript.

## Funding

This study was supported by grants to ZL from the Applied Psychology Research Center of Sichuan Province (CSXL-202A08), Department of Human Resources and Social Security of Sichuan Province [(2020) 291-20], Science and Technology Bureau of Chengdu (2021-YF05-01336-SN), Special Project for Strategic Cooperation between Sichuan University and Dazhou Municipal People’s Government (2022CDDZ-17), and Science and Technology Department of Sichuan Province (2022YFS0349). These funding agencies had no role in the design of the study; collection, analysis, or interpretation of the data; or writing of the manuscript.

## Conflict of interest

The authors declare that the research was conducted in the absence of any commercial or financial relationships that could be construed as a potential conflict of interest.

## Publisher’s note

All claims expressed in this article are solely those of the authors and do not necessarily represent those of their affiliated organizations, or those of the publisher, the editors and the reviewers. Any product that may be evaluated in this article, or claim that may be made by its manufacturer, is not guaranteed or endorsed by the publisher.
